# LRU-Net: lightweight and multiscale feature extraction for localization of ACL tears region in MRI images

**DOI:** 10.3389/fphys.2025.1611267

**Published:** 2025-07-15

**Authors:** Xiaojun Si, Liang Yan, Cui Shi, Yang Xu

**Affiliations:** ^1^ Department of Information Center, Affiliated Hospital of Nantong University, Nantong, China; ^2^ Department of Orthopedics, Nantong Rici Hospital Affiliated to Yangzhou University, Nantong, Jiangsu, China; ^3^ Department of Respiratory Medicine, Nantong Rici Hospital Affiliated to Yangzhou University, Nantong, China

**Keywords:** ACL (anterior cruciate ligament), MRI image, deep learning, segmenation, attention, lightweight

## Abstract

**Introduction:**

Anterior cruciate ligament (ACL) injuries hold significant clinical importance, making the development of accurate and efficient diagnostic tools essential. Deep learning has emerged as an effective method for detecting ACL tears. However, current models often struggle with multiscale and boundary-sensitive tear patterns and tend to be computationally intensive.

**Methods:**

We present LRU-Net, a lightweight residual U-Net designed for ACL tear segmentation. LRU-Net integrates an advanced attention mechanism that emphasizes gradients and leverages the anatomical position of the ACL, thereby improving boundary sensitivity. Furthermore, it employs a dynamic feature extraction module for adaptive multiscale feature extraction. A dense decoder featuring dense connections enhances feature reuse.

**Results:**

In experimental evaluations, LRU-Net achieves a Dice Coefficient Score of 97.93% and an Intersection over Union (IoU) of 96.40%.

**Discussion:**

It surpasses benchmark models such as Attention-Unet, Attention-ResUnet, InceptionV3-Unet, Swin-UNet, Trans-UNet and Rethinking ResNets. With a reduced computational footprint, LRU-Net provides a practical and highly accurate solution for the clinical analysis of ACL tears.

## 1 Introduction

ACL injuries are among the most common and debilitating knee issues, particularly affecting athletes and active individuals (Boden et al., 2000). The ACL plays a crucial role in stabilizing the knee joint by connecting the femur to the tibia, helping to prevent excessive forward movement of the tibia and stabilizing rotation. This ligament is prone to tears during high-impact activities such as pivoting, jumping, or sudden stops (Mattacola et al., 2002), with an estimated annual incidence of 68 per 100,000 person-years in the general population. ACL tears can lead to significant complications, including joint instability, cartilage deterioration, and an increased risk of osteoarthritis, creating significant obstacles to patient mobility and overall quality of life (Dienst et al., 2002; van der List et al., 2017). Therefore, timely and accurate diagnosis of ACL injuries is essential for guiding treatment approaches, ranging from conservative rehabilitation to surgical reconstruction, helping alleviate chronic issues (van der List and DiFelice, 2016; 2018).

Magnetic resonance imaging (MRI) has become the gold standard for non-invasive assessment of ACL injuries, offering superior soft-tissue contrast and multiplanar imaging compared to other methods such as X-rays or ultrasounds (Noone et al., 2005). However, diagnosing ACL tears via MRI can be challenging due to the small size of the ligament (typically 5–10 mm in width), its varying alignment across imaging planes, and the subtle signal changes that indicate partial or complete tears. Although manual interpretation by radiologists is generally practical, it is labor-intensive and subject to variability between observers, with reported sensitivities and specificities ranging from 85% to 95%, depending on the clinician’s experience (Thomas et al., 2007; Sivakumaran et al., 2021). These challenges have led to the development of automated detection methods, particularly those utilizing deep learning techniques to enhance diagnostic accuracy and speed (Zhao et al., 2022).

Deep learning based method have show significantly enhance in classification and segmentation tasks. convolutional neural networks (CNNs) (Krizhevsky et al., 2017) Frameworks such as Attention-Unet (Oktay et al., 2018), and Attention-ResUnet (Li et al., 2022) demonstrated remarkable accuracy on datasets like Imagenet (Deng et al., 2009). Some methods showed special capabilities, especially in tasks of knee joint and skin images (Iqbal et al., 2020; 2021). The architectures of ResNet and UNet have also been continuously improved and optimized, like InceptionV3-Unet (Punn and Agarwal, 2020), Swin-Unet (Cao et al., 2023), Trans-Unet (Chen et al., 2021) and Rethinking ResNets (Luo et al., 2022), However, challenges persist, including high computational demands (e.g., models like Attention-Unet can have over 19 million parameters), insufficient boundary precision in segmentation, and a reliance on advanced hardware that may not be feasible for clinical applications. Additionally, the intricate boundaries of ACL tears in MRI images require models that can effectively identify and delineate injuries.

To address these shortcomings, we propose an LRU-Net that enhances the original design with a lightweight residual encoder utilizing depthwise separable convolutions (Chollet, 2017), a U-Net decoder featuring skip connections, and advanced modules, including optimized dynamic ASPP (Chen et al., 2018) and enhanced lite CBAM (Woo et al., 2018). With a reduced parameter count of 9.1 million, the model employs a hybrid loss function that combines Dice, focal (Lin et al., 2017), and an enhanced boundary term to improve edge precision. Our contributions include:

1. An efficient architecture incorporating depthwise separable ResBlocks in the encoder and U-Net upsampling in the decoder.
2. A multiscale, boundary-aware mechanism through optimized dynamic ASPP and enhanced lite CBAM.
3. Evaluating the proposed LRU-Net method using actual image slices and their corresponding mask slices representing knee ACL tears, along with a comprehensive assessment of the model’s performance and stability.
4. Demonstrating superior capability in localizing the knee ACL tear region in MR images compared to other state-of-the-art methods, including Attention-Unet, Attention-ResUnet, InceptionV3-Unet, Swin-UNet, Trans-UNet and Rethinking ResNets.
5. This study analyzed the impact of various hyperparameters on the performance of the LRU-Net method, offering an in-depth examination of the model’s robustness capabilities.

## 2 Materials and methods

### 2.1 Dataset description

The Affiliated Hospital of Nantong University provided 706 individuals aged 14–81 years who showed clinical evidence of an acute unilateral anterior cruciate ligament (ACL) tear. MRI scans were conducted using 1.5T and 3.0 T modalities. The imaging protocol included an axial T1-weighted fast spin-echo (FSE) sequence for detailed anatomical visualization, a sagittal T2 fat-suppressed (FS) FSE sequence for assessing bone marrow lesions (BML), and sagittal and coronal proton-density (PD)-weighted FSE sequences (Miller, 2009). These PD-weighted sequences were crucial for confirming ACL and other ligament or meniscal injuries and evaluating articular cartilage.

The dataset used in this study consisted of PD-weighted MRI scans of the knee joint, primarily focusing on ACL tears. These scans had an original resolution of 512 × 512 pixels. The images were obtained using a standard PD-weighted protocol, which enhanced contrast for soft tissue structures, particularly the ACL.

### 2.2 Data preparation

The dataset preparation involved a multi-step process to convert raw DICOM images (Mantri et al., 2022) into a suitable format for deep learning analysis, followed by expert annotation (Table 1). The detailed workflow is outlined below:

Step 1: Loading and selecting DICOM images. PD-weighted MRI images, 512 × 512 pixels, were extracted from DICOM files. Expert radiologists selected key slices with ACL tears, including only clinically significant images, thereby reducing noise from irrelevant areas and focusing on ACL tear pathology.
Step 2: Converting DICOM images and generating initial masks. The selected DICOM images were converted to NIFTI format using a custom Python script, maintaining their original resolution. Initial binary mask files in NIFTI format were created and initialized as black (value = 0) to indicate regions without tears. This conversion ensured compatibility with neuroimaging tools and deep learning frameworks.
Step 3: Expert annotation using ITK-SNAP (Yushkevich and Gerig, 2017). The converted NIFTI images and initial masks were loaded into ITK-SNAP, where expert radiologists delineated ACL tear regions, marking them as white (value = 1). The annotated masks replaced the initial ones, producing the final ground truth masks and ensuring high-quality, validated annotations.
Step 4: Image Resizing. For our LRU-Net method, the knee images and masks were resized to 256 × 256 pixels, balancing computational efficiency with detail preservation. Resizing was performed using standard image processing libraries to preserve essential features despite the reduced size.

**TABLE 1:** Algorithm for knee ACL tear mask region extraction with ROI.

Step	Procedure
1	Selection and Loading
	1.1 Extract PD-weighted DICOM images (initial resolution: 512 × 512 pixels) 1.2 Select ACL tear-related slices by expert radiologists
2	Conversion and Initialization
	2.1 Convert DICOM to NII format using Python (maintains 512 × 512 resolution) 2.2 Generate initial black masks in NII format (background value: 0)
3	Annotation with ITK-SNAP
	3.1 Load images and masks into ITK-SNAP 3.2 Annotate ACL tear regions as white by experts (tear regions: value 1) 3.3 Save final masks at 512 × 512 resolution
4	Preprocessing and Augmentation
	4.1 Resize images and masks to 256 × 256 pixels (bilinear interpolation) 4.2 Apply random transformations and ACL tear-specific simulations (flipping, rotation ±1°, brightness ±0.01, contrast ±0.1, tear simulations)

### 2.3 Proposed model architecture

**FIGURE 1:**
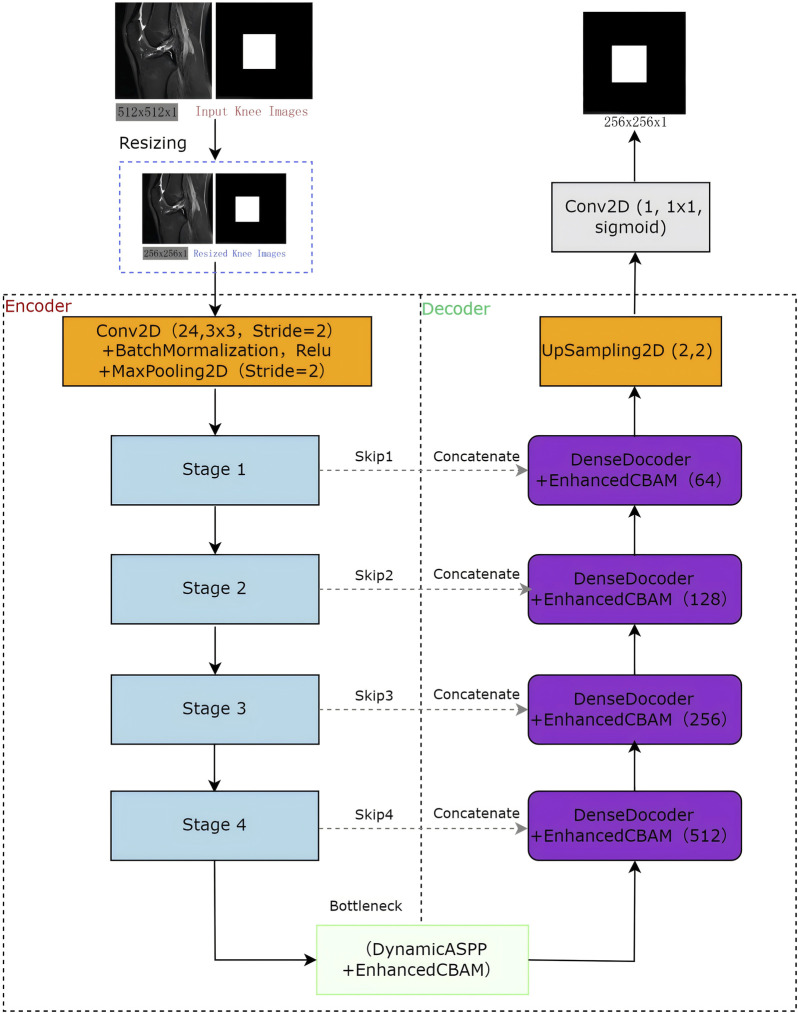
The Knee ACL region location architecture of LRU-Net.

The LRU-Net is a deep learning framework designed for accurate ACL tear segmentation. It combines a lightweight residual encoder with a U-Net-style decoder, enhanced by advanced feature extraction and attention mechanisms specifically adapted to the characteristics of ACL tears (Figure 1). The encoder downsampled the input through three stages to capture hierarchical features. At the bottleneck, the optimized dynamic ASPP and enhanced lite CBAM modules facilitate multiscale context aggregation, refining feature maps to enhance boundary sensitivity. The dense decoder restores spatial resolution across four stages, utilizing dense connections and dropout to improve feature reuse. A 1 × 1 convolutional layer with sigmoid activation generates the final segmentation mask. Optimized for lightweight computation, LRU-Net surpasses traditional models by leveraging anatomical constraints and attention mechanisms.

### 2.4 Encoder-decoder architecture

#### 2.4.1 Encoder

**FIGURE 2:**
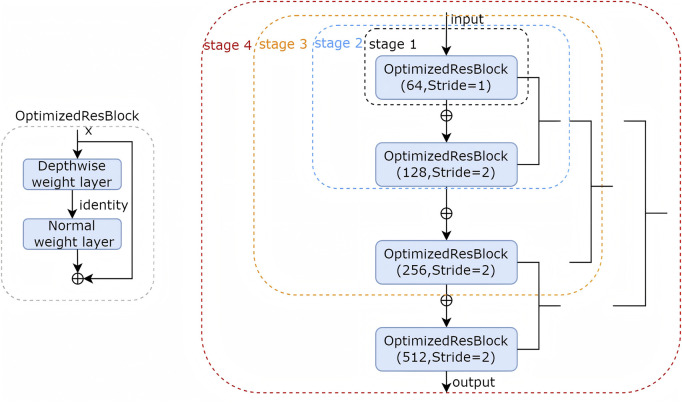
The encoder stages detail.

The encoder employs a hierarchical architecture comprising four stages (stage 1: skip1, stage 2: skip2, stage 3: skip3, stage 4: skip4), each stage utilized optimized ResBlock to progressively downsample spatial resolution, extracting increasingly abstract feature representations (Figure 2). In the stage 2, stage 3 and stage 4 we use mid-point addition to expand the channel depth progressively and facilitate the integration of low and high-level features. the formula of mid-point addition is:

$$X_{\text{mid}}=X_{\text{in}}+\frac{1}{2}K\;\left(K=f\left(X_{\text{in}}\right)\right)$$

$$X_{\text{out}}=f\left(X_{\text{mid}}+X_{\text{in}}\right)+X_{\text{mid}}$$

There were two noteworthy differences in every stage: (1). when adding the shortcut, the mid-point design compressed the output of symbol by half. (2). The second shortcut is from an earlier location, which is directly from input. By using this mid-point method, the number of parameters in LRU-Net is less than that in the conventional ResBlock. The optimized ResBlock integrates two weight layers, which combining efficient depthwise separable convolutional operations, 1 × 1 pointwise convolutions for channel reduction and expansion with 3 × 3 depthwise convolutions for spatial feature extraction, alongside batch normalization and ReLU activation. achieving superior computational efficiency characteristic of lightweight convolutional designs. The encoder effectively captures multiscale features, encompassing fine-grained details, such as tear textures, and broader anatomical contexts.

#### 2.4.2 Bottleneck

At the bottleneck, the optimized dynamic ASPP employs dynamic dilation rates for multiscale feature integration. This is followed by an enhanced lite CBAM that incorporates channel, gradient, spatial, and ACL position prior attention, refining the features for a tear-specific focus.

#### 2.4.3 Decoder

The decoder upsampled features using four dense decoder blocks, each enhanced by lite CBAM, along with skip connections (skip1, skip2, skip3 and skip4) to refine tear boundaries. Additional UpSampling2D layers restore the original resolution, and a 1 × 1 Conv2D with sigmoid activation produces the final segmentation mask.

### 2.5 Attention mechanism

The LRU-Net employs two distinct attention mechanisms to enhance its segmentation performance. These mechanisms are strategically integrated into the bottleneck and decoder stages, utilizing multiscale feature extraction and task-specific feature refinement to improve recognition capability and boundary segmentation accuracy.

#### 2.5.1 Attention mechanism I

**FIGURE 3:**
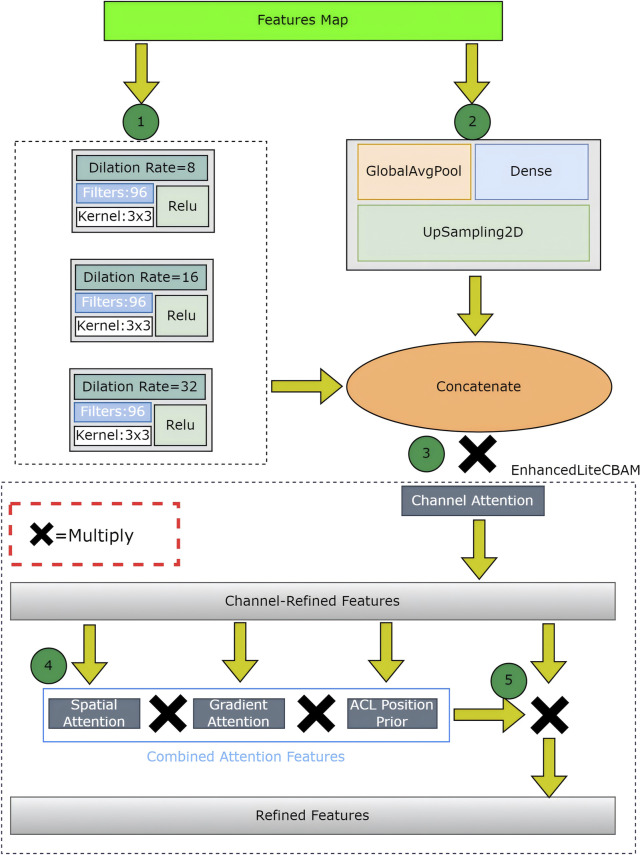
The Attention Mechanism I detail.

The first attention mechanism at the bottleneck combines optimized dynamic ASPP and enhanced lite CBAM to process the encoder’s output, producing a refined feature map. Optimized dynamic ASPP dynamically adjusts dilation rates (e.g., 8, 16, 32), utilizing parallel convolutions and a global context branch (GlobalAvgPool, Dense, UpSampling2D) to capture multiscale features, which are then fused via a 1 × 1 Conv2D layer. Enhanced lite CBAM refines these features through channel, spatial, and gradient attention (via sobel filters) and an ACL position prior (Si et al., 2025), which prioritizes tear-relevant regions and reduces false positives (Figure 3). This mechanism enhances boundary segmentation accuracy, with optimized dynamic ASPP providing robust multiscale context and enhanced lite CBAM improving sensitivity to acceptable tear boundaries, achieving a low boundary dice loss.

#### 2.5.2 Attention mechanism II

**FIGURE 4:**
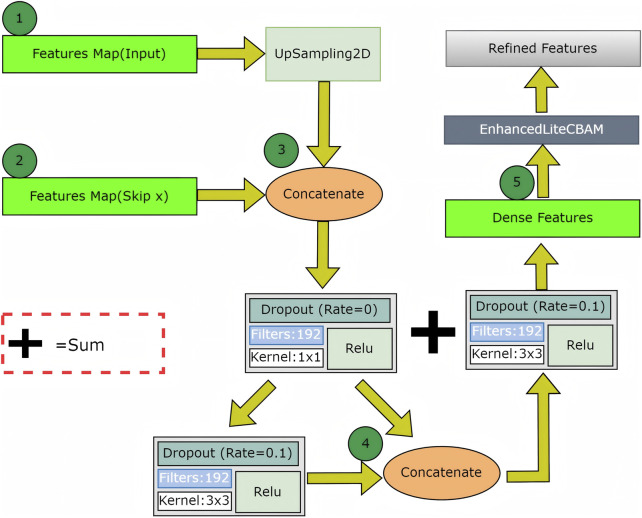
The Attention Mechanism II detail.

The second attention mechanism in the decoder employs dense blocks with enhanced lite CBAM across four skip connections, improving feature recovery and boundary refinement for the final segmentation mask (Figure 4). The dense decoder up-samples the bottleneck output using UpSampling2D layers and concatenates features from the encoder. Dense connections and dropout enhance feature reuse and prevent overfitting, followed by convolutional layers for processing. This module recovers spatial details from skip connections. After each dense block, enhanced lite CBAM refines features through channel, gradient, spatial attention, and ACL position prior, emphasizing tear boundaries and relevant regions. It preserves tear-relevant features across scales, enhancing the recognition of complex tear patterns. By integrating these modules, superior boundary segmentation accuracy is achieved. The dense decoder ensures feature consistency for smooth transitions at tear boundaries, while enhanced lite CBAM highlights fine edges and the ACL position, resulting in precise tear delineations with minimal errors.

### 2.6 Experimental setup

This study was conducted on a 12th-generation Intel Core i7 processor with 12 cores and 20 threads, 32 GB of RAM, and an NVIDIA GeForce RTX 3080 GPU with 8960 CUDA cores. The software environment included PyCharm Professional as the integrated development environment (IDE), with Python 3.9.12 as the programming language. The TensorFlow 2.10.0 and Keras 2.10.0 packages were utilized for model development and training. This consistent hardware and software configuration ensured fairness and reproducibility across all experiments.

### 2.7 Training and validation split

The dataset was divided into training and validation subsets using an 80:20 ratio to ensure robust evaluation and prevent overfitting. The division, implemented in the data loading function with a random shuffle (buffer size 1000), ensured a representative distribution of tear patterns. The shuffle method, seeded by the system clock, guaranteed reproducibility. The validation set was used solely for performance monitoring, ensuring no data leakage. Both datasets were batched (size 8), resulting in 40 training steps and 10 validation steps per epoch. Early stopping (patience of 10 epochs) was employed to monitor the validation dice coefficient score and optimize model selection. Through data augmentation, this strategy enabled a comprehensive assessment of LRU-Net’s ACL tear segmentation across diverse imaging conditions.

### 2.8 Evaluation metrics

A range of metrics was utilized to thoroughly evaluate the performance of the LRU-Net for ACL tear segmentation, focusing on both pixel-wise accuracy and region-based overlap. These metrics were calculated on the training and validation sets during each epoch, offering valuable insights into the model’s segmentation accuracy, boundary precision, and generalization ability. The evaluation metrics applied in this study include Accuracy, F1 Score, Intersection over Union (IoU), Dice Coefficient Score, Dice Loss, and Boundary Dice Loss, described in detail as follows:

(a) Accuracy: This metric evaluates the proportion of correctly classified pixels throughout the image, defined as:
Accuracy = (Correctly predicted tear pixels + Correctly predicted non-tear pixels)/Total number of pixels.
(b) F1 Score: The F1 Score measures the balance between precision and recall in detecting tear regions. Precision (P) and recall (R) are calculated as follows:
P = Correctly predicted tear regions/(Correctly predicted tear regions + Incorrectly predicted tear regions)
R = Correctly predicted tear regions/(Correctly predicted tear regions + Actual tear regions incorrectly identified as non-tear regions)
Thus, the F1 Score is computed as:

$$F1=\left(P+R\right)\;/\;\left(2\times P\times R\right)$$

(c) Intersection over Union (IoU): IoU quantifies the overlap between predicted and actual tear regions, defined as:
IoU = Correctly predicted tear regions/(Correctly predicted tear regions + Incorrectly predicted tear regions + Actual tear regions incorrectly identified as non-tear regions)
(d) Dice Coefficient Score (DCS): The DCS measures the likeness between the predicted and actual tear regions in the ground-truth mask, defined as:
DCS = 2 × Correctly predicted tear regions/(Total predicted tear regions + Total actual tear regions)
(e) Dice Loss: The Dice loss of the LRU-Net combines the Dice-frequency boundary loss (DBL) to enhance segmentation accuracy, boundary precision, and resilience to class imbalance. The Dice Loss is defined as:
DL = 1 - [2 × Correctly predicted tear regions/(Total predicted tear regions + Total actual tear regions)]
BL = 1 - [2 × Correctly predicted boundary tear regions/(Total predicted boundary tear regions + Total actual boundary tear regions)]
(f) Boundary Dice Loss (BDL): The Boundary Dice Loss assesses the model’s capacity to accurately trace tear boundaries based on training loss. Using the distance transform method, it prioritizes edge regions by applying a boundary weight factor of 5. The BDL is defined as:
BDL = 1 - [2 × Correctly predicted tear regions/(Total predicted tear regions + Total actual tear regions)]

**FIGURE 5:**
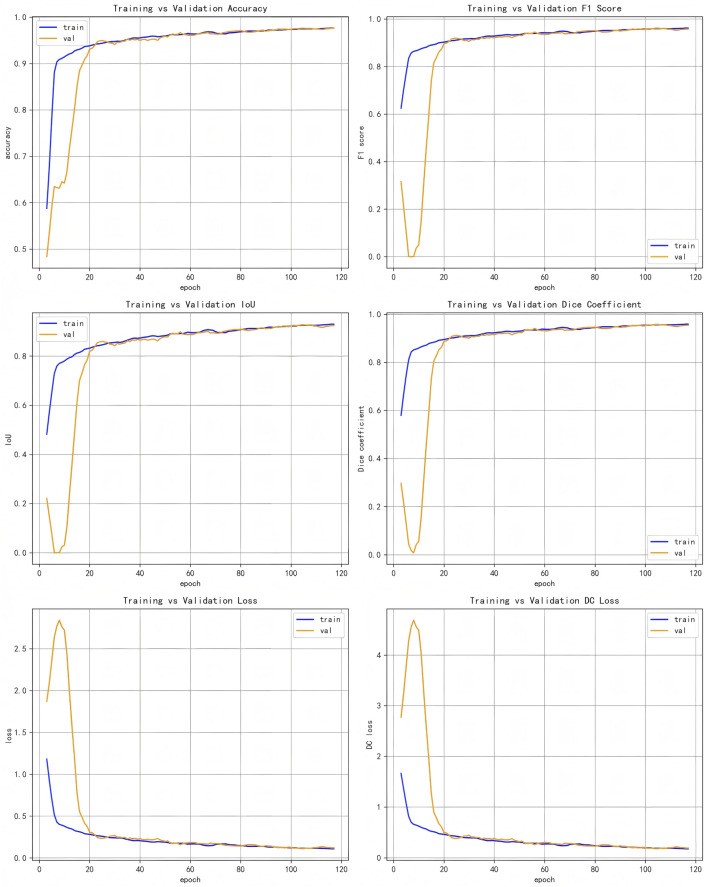
Evaluation metrics plots of training vs validation data set.

In the formulas, “Correctly predicted tear regions” refers to true positives (TP), “Incorrectly predicted tear regions” corresponds to false positives (FP), “Total predicted tear regions” represents the sum of true positives (TP) and false positives (FP), “Actual tear regions incorrectly identified as non-tear regions” corresponds to false negatives (FN), and “Total actual tear regions” refers to the sum of true positives (TP) and false negatives (FN).

(Figure 5) displays the accuracy plots against validation data for our proposed model’s evaluation metrics and loss values, illustrating different evaluation metrics as a function of training epochs on both validation and training data for the LRU-Net method.

## 3 Results

This section presents the experimental results of the LRU-Net for ACL tear segmentation on a dataset of MRI images. The results are evaluated using multiple metrics as defined in Section 2.8. We report quantitative and qualitative outcomes and compare the model’s performance against baseline models, including Attention-Unet, Attention-ResUnet, InceptionV3-Unet, Swin-UNet, Trans-UNet and Rethinking ResNets. All metrics are calculated using the training and validation sets (Table 2). Highlights the performance of our method, LRU-Net, which achieved the highest accuracy, F1 score, IoU, Dice Coefficient Score, Dice loss, and Boundary Dice Loss at 98.83%, 98.16%, 96.40%, 97.93%, 5.25%, and 8.60% on the validation data.

**TABLE 2:** The evaluation metric score comparison of our method, LRU-Net, with six other methods on the training and validation sets.

Method	Evaluation metrics score on training data						Evaluation metrics score on validation data					
	Accuracy	F1 Score	IoU	Dice Coefficient Score	Dice Loss	Boundary Dice Loss	Accuracy	F1 Score	IoU	Dice Coefficient Score	Dice Loss	Boundary Dice Loss
LRU-Net	**98.87%**	**97.58%**	**96.45%**	**98.71%**	**5.13%**	**8.19%**	**98.83%**	**98.16%**	**96.40%**	**97.93%**	**5.25%**	**8.60%**
Attention-Unet	94.11%	90.61%	83.76%	90.19%	26.0%	44.22%	94.67%	90.72%	83.75%	90.10%	26.36%	42.91%
Attention-ResUnet	95.75%	93.06%	89.72%	92.51%	20.2%	19.70%	95.97%	93.38%	88.14%	92.38%	19.70%	31.88%
InceptionV3-Unet	97.67%	95.92%	89.66%	95.82%	11.1%	18.71%	97.75%	96.40%	93.20%	97.82%	10.48%	17.13%
Swin-Unet	96.88%	95.33%	91.29%	95.71%	11.3%	16.65%	97.03%	96.17%	91.17%	96.21%	13.54%	19.97%
Trans-Unet	98.16%	97.01%	95.28%	97.97%	7.02%	9.72%	97.95%	97.53%	95.82%	96.81%	9.33%	10.04%
Rethinking ResNets	98.70%	97.36%	96.30%	98.53%	6.07%	9.27%	98.73%	97.98%	96.33%	97.36%	5.81%	9.77%

The bold values highlights that our proposed method LRU-Net achieved the highest accuracy, F1 score, IoU, Dice Coefficient Score, Dice loss, and Boundary Dice Loss on the validation data.

**FIGURE 6:**
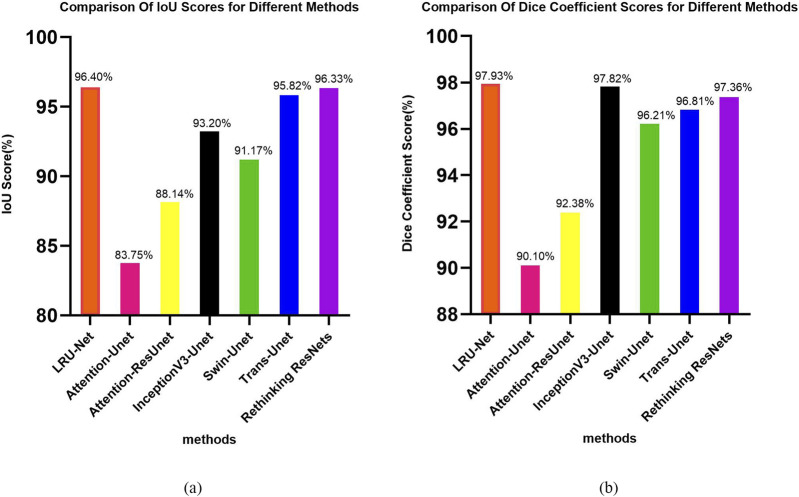
**(a,b)** The IoU and dice coefficient score plot of all methods.

In both the Dice Coefficient Score figure and the IOU plot, the proposed LRU-Net method demonstrated exceptional performance, achieving the highest scores among all evaluated methods (Figures 6a,b).

**FIGURE 7:**
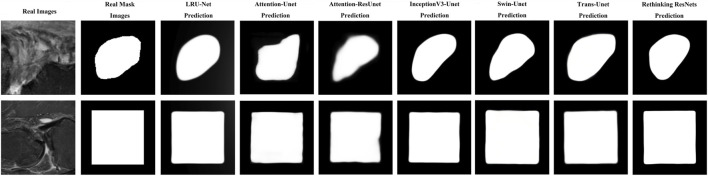
Example images of LRU-Net comparison with other methods results of real, ground truth, and predicted mask region location.

As illustrated in Figure 7, the result indicated the real images in the first column. The second column is the ground truth masking of the ACL tear region. The third column is the result of our proposed LRU-Net. The other columns are the predicted results produced by other various models.

It showcases sample real images of ACL tear regions, the corresponding ground truth masks, the results obtained using our proposed LRU-Net, and the predicted results produced by the six other models. It offers a visual comparison of the performance of each model in localization ACL tear regions and demonstrates the effectiveness of LRU-Net in comparison to other models. The proposed LRU-Net method has the least overfitting as the difference between the training and test loss values is small. The Rethinking ResNets also have relatively low overfitting. Additionally, the proposed method also has a low number of trainable parameters compared to other models with the same encoder layers and skip connections (Table 3), which may have contributed to its high performance while avoiding overfitting. On the other hand, the Attention_Unet and Attention_ResUnet models seem to underfit as they have low test scores and high training loss values.

**TABLE 3:** Parameters comparison of LRU-Net and the other six methods.

Method	Parameters (M)
LRU-Net	9.1 M
Attention-Unet	19.15 M
Attention-ResUnet	19.7 M
InceptionV3-Unet	10.2 M
Swin-Unet	11.2 M
Trans-Unet	13.4 M
Rethinking ResNets	15.8 M

The significant improvement in localization accuracy highlights the effectiveness of the LRU-Net method in accurately identifying and delineating ACL tears. These findings validate the superiority of our proposed method and its potential for enhancing the diagnosis and treatment of ACL injuries.

## 4 Discussion

An ablation study was conducted by training and testing four variants of the LRU-Net, each with specific components either removed or modified, to evaluate their respective contributions. The components investigated were the optimized dynamic ASPP module, the enhanced lite CBAM attention mechanism, and dense connections in the dense decoder (Table 4). All variants were trained and evaluated under the same experimental setup for consistency.

**TABLE 4:** Ablation study on the impact of four components in LRU-Net.

Ablation study	Accuracy	F1 score	IoU	Dice coefficient Score	Dice loss	Boundary dice loss
Without Attention Mechanisms	92.67%	88.70%	80.64%	88.03%	32.20%	54.22%
Without Optimized dynamic ASPP	95.72%	93.08%	87.83%	92.86%	19.10%	32.73%
Without Enhanced lite CBAM	96.88%	94.90%	90.58%	94.02%	15.92%	23.79%
Without Dense Connections	95.91%	93.48%	88.29%	93.23%	18.05%	30.93%

### 4.1 LRU-Net without attention mechanisms

Excluding both optimized dynamic ASPP and enhanced lite CBAM (by replacing optimized dynamic ASPP with a Conv2D layer) resulted in a significant performance drop across all metrics, highlighting the synergistic role of these mechanisms in capturing multiscale features and refining tear-relevant information for high segmentation accuracy and boundary precision.

### 4.2 LRU-Net without optimized dynamic ASPP

Removing optimized dynamic ASPP (replaced with a Conv2D layer) reduced performance, underscoring its importance in dynamic multiscale feature extraction for tears of varying sizes.

### 4.3 LRU-Net without enhanced lite CBAM

Excluding enhanced lite CBAM decreased all metrics, indicating its critical role in focusing on tear-relevant features and enhancing boundary delineation.

### 4.4 LRU-Net without dense connections

Replacing dense connections with standard skip connections in the dense decoder reduced performance, confirming their role in maintaining feature consistency and improving boundary transitions.

The ablation study highlights the critical contributions of optimized dynamic ASPP, enhanced lite CBAM, and dense connections to the performance of LRU-Net. Excluding optimized dynamic ASPP and enhanced lite CBAM resulted in a significant performance drop across all metrics, underscoring their synergistic role in capturing multiscale features and refining tear-relevant information for high segmentation accuracy and boundary precision. Removing optimized dynamic ASPP alone reduced performance, emphasizing its importance in dynamic multiscale feature extraction for varying-sized tears. Similarly, excluding enhanced lite CBAM decreased all metrics, indicating its essential role in focusing on tear-relevant features and enhancing boundary delineation. Replacing dense connections with standard skip connections in the dense decoder also lowered performance, confirming their role in maintaining feature consistency and improving boundary transitions. These findings validate the necessity of each component for effective segmentation of ACL tears. Compared to baselines such as U-Net and Res-Unet, LRU-Net’s lightweight design and superior metrics highlight its efficiency for clinical deployment. Clinically, LRU-Net’s precision aids accurate tear localization, supporting surgical planning and reducing diagnostic variability. The primary contributions of this work lie in the two synergistic attention mechanisms embedded within the LRU-Net. Attention Mechanism I, which combines optimized dynamic ASPP and enhanced lite CBAM at the bottleneck, captures multiscale contextual features and refines them with gradient and anatomical attention. It enables the model to recognize tear patterns across varying scales while enhancing boundary sensitivity. Attention Mechanism II, which integrates the dense decoder and enhanced lite CBAM across skip connections, ensures precise feature recovery and boundary refinement during decoding. It leverages dense connections and attention mechanisms to maintain feature consistency and focus on tear-relevant regions. These mechanisms collectively contribute to the model’s ability to achieve high segmentation accuracy and boundary precision, as evidenced by the low boundary dice loss and qualitative improvements in tear boundary delineation compared to baselines.

Despite its strong performance, the model has certain limitations. The dataset size (706 individuals) may limit its ability to capture rare or extreme tear patterns, potentially restricting generalization in diverse clinical scenarios. Additionally, while the boundary dice loss of 5.25% is low, there remains room for further improvement in boundary precision, particularly for tears with extremely low contrast or complex shapes. Future research could address these limitations by expanding the dataset through synthetic data generation or multi-center data collection to enhance model robustness and accuracy. Moreover, incorporating advanced edge-preserving techniques, such as conditional random fields (CRFs) (Krähenbühl and Koltun, 2012), as a post-processing step could further refine boundary segmentation. Exploring hybrid architectures that combine the dense connections of DenseNet (Huang et al., 2017) with the attention mechanisms of LRU-Net may also yield additional improvements in feature reuse and segmentation accuracy.

## 5 Conclusion

This study introduced the LRU-Net, a deep-learning framework designed to precisely segment anterior cruciate ligament (ACL) tears in MRI images. By integrating lightweight residual blocks, dynamic multiscale feature extraction, and task-specific attention mechanisms, the model addresses the challenges of multiscale tear patterns, delicate and irregular boundaries, and anatomical variability in clinical MRI data. The experimental results demonstrate the model’s superior performance. The LRU-Net represents a significant advancement in ACL tear segmentation, leveraging innovative attention mechanisms to achieve high accuracy and boundary precision. Its lightweight design, robust performance, and clinical relevance underscore its potential for practical deployment in medical imaging applications, paving the way for future research in automated diagnostic systems.

## Data Availability

The raw data supporting the conclusions of this article will be made available by the authors, without undue reservation.
